# Luseogliflozin Persistently Lowers Glycemia and Body Weight Over a Period of Two Years in Patients With Type 2 Diabetes

**DOI:** 10.7759/cureus.78503

**Published:** 2025-02-04

**Authors:** Jun-Ichirou Mori, Koh Yamashita, Toru Aizawa

**Affiliations:** 1 Center for Medical Education, Faculty of Medicine, Shinshu University, Matsumoto, JPN; 2 Diabetes Center, Aizawa Hospital, Matsumoto, JPN

**Keywords:** body mass index: bmi, body weight, hemoglobin a1c, sodium-glucose co-transporter 2 inhibitor, type 2 diabetes

## Abstract

Introduction: Sodium-glucose co-transporter-2 inhibitor (SGLT2i) was introduced as an oral hypoglycemic agent. Thereafter, it was revealed that SGLT2i had multifaced effects, such as renal function and heart failure. Luseogliflozin is one of the SGLT2i. However, the long-term effects of luseogliflozin on glycemic control, body weight, and body mass index have not yet been established.

Aims: This study aimed to determine the long-term effects of luseogliflozin, an SGLT2i, on plasma glucose (PG), body weight (BW), body mass index (BMI), and blood pressure (BP) in patients with type 2 diabetes.

Methods: Luseogliflozin was administered continuously in an outpatient clinic for at least two years to 35 SGLT2i-naive patients. Random plasma glucose (RPG), hemoglobin A1c (HbA1c) level, BW, BMI, and BP were measured every six months.

Results: At baseline, the patients had a median age of 63 years and a median diabetes duration of seven years. The mean RPG levels at the initiation of luseogliflozin and after 1 and 2 years were 213.5, 155.7, and 164.4 mg/dL, respectively. The reductions from baseline at both 1 year (p<0.001) and 2 years (p=0.012) were statistically significant. Compared with the baseline, the mean HbA1c level significantly decreased by 0.98% after 1 year (p<0.001) and by 1.08% after 2 years (p<0.001), respectively. The mean BMI significantly decreased from 27.41 kg/m² at baseline to 26.53 kg/m² after 1 year (p<0.001) and 26.37 kg/m² after 2 years (p<0.001). Systolic/diastolic blood pressure, initially (138.2/79.2 mmHg), was reduced to 132.9/74.5 mmHg after 1 year, with statistically significant declines in both systolic (p=0.035) and diastolic (p=0.016) pressure compared to baseline.

Conclusions: The effects of luseogliflozin on glucose and BW reduction were sustained for at least two years, indicating similarity to other SGLT2 inhibitors.

## Introduction

Treatment of type 2 diabetes (T2D) has undergone considerable changes over the past quarter-century. Insulin analogs with various durations of action have been developed since 2000; in 2010, a glucagon-like peptide-1 (GLP-1) receptor agonist (GLP-1 RA) became available in Japan. Oral hypoglycemic agents (OHAs) such as alpha-glucosidase inhibitors, short-acting insulin secretagogues (glinides), and thiazolidinedione (TZD) were launched in the 1990s, which ended the long era of sulfonylurea (SU) and biguanide (BG) occupation of the market that began in the 1950s. Recently, dipeptidyl peptidase-4 inhibitors (DPP4i), GLP-1 RA, and sodium-glucose co-transporter-2 inhibitors (SGLT2i) have been marketed, dramatically expanding the options for the treatment of T2D [1].

SGLT2i is the agent that exerts a hypoglycemic effect by excreting glucose in the urine, resulting in a weight loss effect [2]. In other words, this drug has a low risk of hypoglycemia, has a hypoglycemic effect independent of insulin, and can be expected to improve insulin resistance by weight loss. Improved glycemic management is beneficial, especially for the development of microvascular complications [3]. On the other hand, it has been pointed out that patients with type 2 diabetes often have comorbidities such as dyslipidemia and hypertension [4]. Body weight loss itself has been reported to be beneficial for dyslipidemia and hypertension [5,6]. Therefore, SGLT2i is considered beneficial not only in the treatment of type 2 diabetes but also in the treatment of comorbidities.

Among OHAs, SGLT2i appears to uniquely possess many favorable effects beyond simple glucose reduction, including the reduction of cardiovascular (CV) events, CV-related death, non-fatal myocardial infarction, and non-fatal stroke [7], as well as slowing the decline of the estimated glomerular filtration rate (eGFR) and reducing microalbuminuria [8]. It also reduces the risk of heart failure and hospitalization due to heart failure [9]. In addition, it has favorable effects on nonalcoholic fatty liver disease and sleep apnea syndrome [10,11], with additional potential multifaceted benefits still under investigation [12]. However, the use of SGLT2i is relatively recent, and its long-term efficacy and safety have not yet been firmly established.

Luseogliflozin is a unique SGLT2i metabolized by the liver and kidneys, and it can be used in patients with both renal and hepatic dysfunction [13]. However, the long-term effects of luseogliflozin on plasma glucose, body weight (BW), and hemoglobin A1c (HbA1c) levels have not yet been established. We conducted this study to confirm that luseogliflozin has a lasting effect, similar to other SGLT2is, such as empagliflozin and canagliflozin.

## Materials and methods

Participants

**Figure 1:**
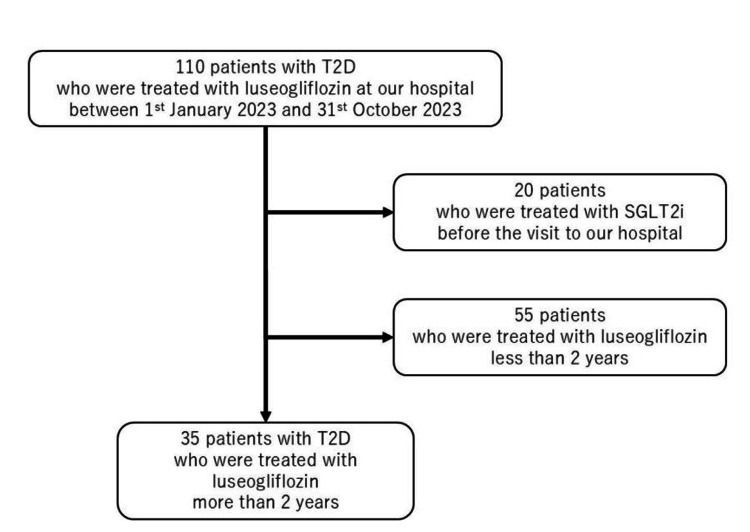
The protocol of the patient selection. At first, 110 patients who were treated with luseogliflozin at our hospital between January 1, 2023, to October 31, 2023, were registered. Twenty patients were excluded due to their previous sodium-glucose cotransporter-2 inhibitor use, and 55 patients were excluded because they had not been treated with luseogliflozin for 2 years. As a result, 35 patients were included in the study.

This retrospective, observational study clarified the long-term effects of luseogliflozin by observing patients who continuously administered the drug for two years. Figure 1 shows the protocol of the patient selection.

Initially, 110 patients with T2D treated with luseogliflozin at our hospital between January 1, 2023, and October 31, 2023, were registered. Diabetes was diagnosed if fasting plasma glucose (FPG)≥126 mg/dL, RPG≥200 mg/dL, and HbA1c≥6.5% or if the patient had already been diagnosed at external clinics or hospitals and was receiving hypoglycemic agents. All patients were instructed on proper diet and exercise. Nonetheless, luseogliflozin was added when the therapeutic effect was insufficient and the therapeutic goal was not reached. Twenty patients were excluded due to previous SGLT2i use, and 55 were excluded because they had not received luseogliflozin for two years. After applying the exclusion criteria, 35 patients with T2D who had received luseogliflozin for ≥2 years were retrospectively and observationally evaluated. Additionally, adherence to luseogliflozin was assessed through self-reported patient feedback.

Anthropometric data

BW, BP, and pulse rate (PR) were recorded from electronic records every six months. The data on BW, BP, and PR for each month were examined at each patient’s visit. BP was measured by the patient himself using an automatic blood pressure monitor HBP-9020 (OMRON, Kyoto, Japan) after 10 minutes of rest. Body mass index (BMI) was calculated by dividing BW (kg) by the square of body height (m^2^).

Laboratory data

RPG was determined by the glucose oxidase method [14] using Arkray Adams GA-1171 (Tokyo, Japan) or Labospect 008 Alpha (Hitachi, Ibaraki, Japan). HbA1c levels were determined using high-performance liquid chromatography [15] with an Arkray HA8181 (Tokyo, Japan). RPG and HbA1c for each time axis were measured promptly by obtaining the blood sample at the time of each patient's visit. The RPG and HbA1c results were collected retrospectively from their electric records.

Statistical analysis

Descriptive statistical analysis was performed using Chi-square or Fisher’s exact tests. Changes over time were examined using Wilcoxon's Signed-Rank test. All analyses were performed at the nominal alpha level of 0.05 without correction for multiple hypothesis testing. No formal power calculations were performed in advance for the changes over time. JMP version 17 (SAS Institute, North Carolina, USA) was used for calculations, with statistical significance set at p<0.05. Data are expressed as means and standard deviations (SD) unless otherwise stated. The standard error (SE) was calculated by dividing the standard deviation of the parameter by the square root of the parameter size.

## Results

Table 1 shows the baseline demographic data of the patients. Of the 35 patients included, 24 were males (68.6%). Patients were aged 63 years with seven years of diabetes. The median BW was 72.6 kg, and the median BMI was 23.91 kg/m^2^. The median values of systolic BP (SBP), diastolic BP (DBP), and PR were 136 mmHg, 76.5 mmHg, and 82 beats per minute (bpm), respectively.

**Table 1:** Baseline demographical data of the patients. Values are expressed as median [inter-quartile range] except for sex and age which were number (%) and median (range).

Parameters	Values
Sex (male)	24 (68.6)
Age (years old)	63.0 (37-83)
Diabetes duration (years)	7.0 [3.0-14.0]
Systolic blood pressure (mmHg)	136.0 [128.3-148.8]
Diastolic blood pressure (mmHg)	76.5 [71.0-82.0]
Heart rate (beats/min)	82.0 [76.0-90.8]
Body weight (kg)	72.6 [62.8-81.6]
Body mass index (kg/m^2^)	23.91 [26.22-29.44]

Table 2 shows the baseline laboratory data of the patients. The median RPG was 207 mg/dL, and HbA1c was 8.4%. The median value of red blood cell count was 479.5 million/µL, hemoglobin (Hgb) was 14.45 g/dL, and hematocrit (Hct) was 42.4%.

**Table 2:** Baseline laboratory data of the patients. Values are expressed as median [inter-quartile range]. AST: aspartate transaminase; ALT: alanine transaminase; GGT: gamma-glutamyl transferase.

Parameters	Values
Plasma glucose concentration (mg/dL)	207.0 [141.0-285.0]
Hemoglobin A1c level (%)	8.40 [7.65-9.45]
Red blood cell count (x10^6/µL)	479.5 [436.-510.1]
Hemoglobin (g/dL)	14.45 [13.48-15.60]
Hematocrit (%)	42.40 [39.73-46.53]
Estimated glomerular filtration rate (mL/min/1.73m^2)	73.14 [56.86-86.85]
AST (IU/L)	30.5 [22.3-62.0]
ALT (IU/L)	43.5 [19.0-78.8]
GGT (IU/L)	50.5 [29.5-103.0]
Fibrosis-4 index	2.070 [1.310-2.785]

Table 3 shows the number of medications administered at the start of luseogliflozin treatment. The median number of medications taken for T2D was two. Only one patient was drug-naive; five were treated with one drug, 22 were treated with two drugs, six were treated with three drugs, and one was treated with four drugs.

**Table 3:** Number of medications for diabetes at the beginning of luseogliflozin.

Number of medications for diabetes	(number, %)
0	1, 2.9
1	5, 14.3
2	22, 62.9
3	6, 17.1
4	1, 2.9
≥5	0, 0.0

Table 4 shows the types of medications administered at the start of luseogliflozin treatment. Before luseogliflozin administration, 20 patients (57.1%) were taking biguanide (BG), eight patients (22.9%) were taking sulfonylurea (SU), two patients (5.7%) were taking glinide, one patient (2.9%) was taking alpha glucosidase-inhibitor (alpha GI), thiazolidinedione (TZD), and glucagon-like polypeptide-1 receptor agonist (GLP-1 RA), 31 patients (88.6%) were taking dipeptidyl peptidase-4 inhibitor (DPP4i), and seven patients were using insulin (20%).

**Table 4:** Number of patients taking each medication before luseogliflozin. BG: biguanide; SU: sulfonylurea; Glinide: insulin secretagogue; Alpha GI: alpha-glucosidase inhibitor; TZD: thiazolidinedione; DPP-4i: dipeptidyl peptidase-4 inhibitor; GLP-1 RA: glucagon-like polypeptide-1 receptor agonist.

Type of medications	(number, %)
BG	20, 57.1
SU	8, 22.9
Glinide	2, 5.7
Alpha GI	1, 2.9
TZD	1, 2.9
DPP-4i	31, 88.6
GLP-1 RA	1, 2.9
Insulin	7, 20.0

**Figure 2:**
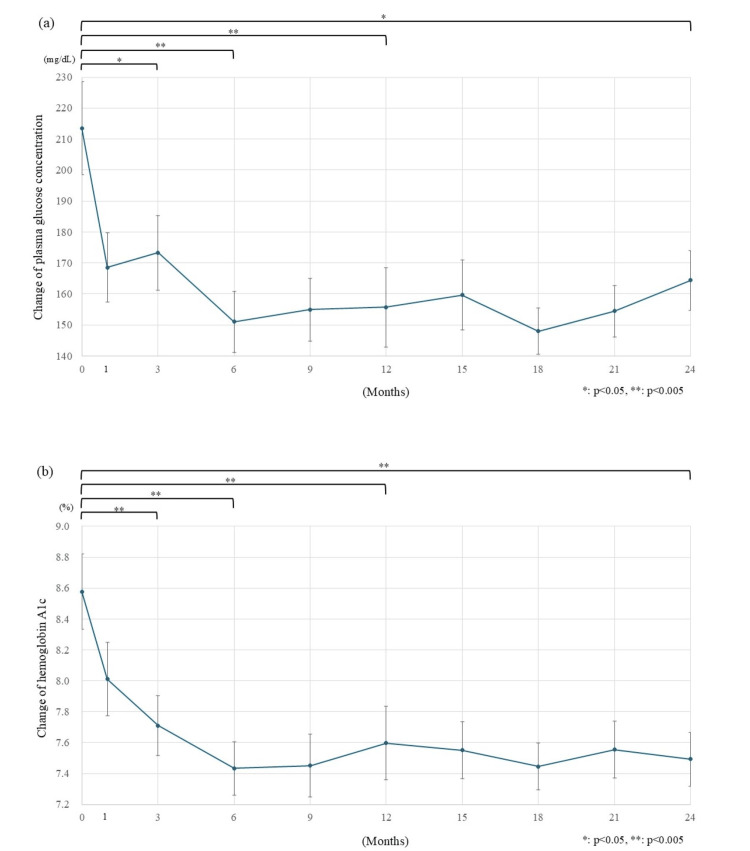
Changes in glucose metabolism. a) Plasma glucose concentration decreased rapidly after the initiation of luseogliflozin treatment. The changes from the baseline of plasma glucose concentration decreased by 57.8 mg/dL (from 213.5 mg/dL to 155.7 mg/dL) after 1 year and 49.1 mg/dL (to 164.4 mg/dL) after 2 years compared to that before the start of treatment. b) HbA1c levels declined rapidly, with the decrease continuing over 2 years. Three months after the start of luseogliflozin administration, the decrease from the baseline in HbA1c levels was 0.87%. The reduction effect was still significantly different two years later. HbA1c: hemoglobin A1c; The error bars show standard error, *: p<0.05; ** p<0.005.

The changes in plasma glucose concentration and the value of HbA1c are shown in Figure 2. The mean concentration of RPG immediately decreased from 213.5 mg/dL to 173.3 mg/dL after three months (-40.2 mg/dL, p=0.006) (Figure 2a). The decline continued to 155.7 mg/dL after 1 year (-57.8 mg/dL) and to 164.4 mg/dL after 2 years (-49.1 mg/dL) following the initiation of luseogliflozin (p<0.001 and p 0.012, respectively). HbA1c also decreased rapidly after the start of luseogliflozin, dropping from 8.58% to 7.71% (-0.87%, p<0.001) three months after starting luseogliflozin to 7.6% (-0.98%, p<0.001) after one year, and kept lowering to 7.49% (-1.09%, p<0.001) after two years (Figure 2b).

**Figure 3:**
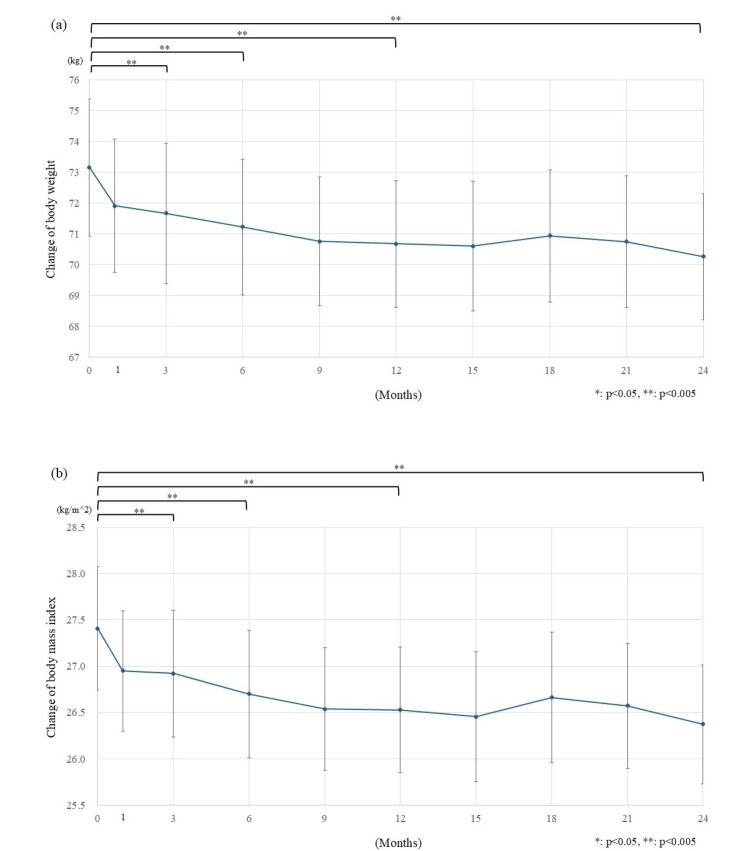
Changes in body weight and body mass index. a) Body weight decreased quickly after the initiation of luseogliflozin treatment. Weight gradually changed by -2.48 kg (from 73.17 kg to 70.69 kg) after 1 year and -2.90 kg (to 70.27 kg) after 2 years. b) Body mass index showed a rapid decline, with the reduction effect remaining significant after two years. The decrease from the baseline was 0.88 kg/m^2^ (from 27.41 kg/m^2^ to 26.53 kg/m^2^) after 1 year and 1.04 kg/m^2^ (to 26.37 kg/m^2^) after two years. The error bars show standard error. *: p<0.05; **: p<0.005

The changes in BW and BMI are shown in Figure 3. BW decreased by 1.50 kg (from 73.17 kg to 71.67 kg) in the first three months (p<0.001) (Figure 3a). The BW decreased to 70.69 kg (-2.48 kg) 1 year after the start of luseogliflozin and to 70.27 kg (-2.90 kg) 2 years after the start of luseogliflozin (p<0.001 and p<0.001, respectively). Changes in BMI were statistically significant at p<0.001, from 27.41 kg/m^2^ to 26.92 kg/m^2^ (-0.49 kg/m^2^) after three months, at p<0.001 to 26.53 kg/m^2^ (-0.88 kg/m^2^) after 1 year, and at p<0.001 to 26.37 kg/m^2^, (-1.04 kg/m^2^) after 2 years (Figure 3b).

**Figure 4:**
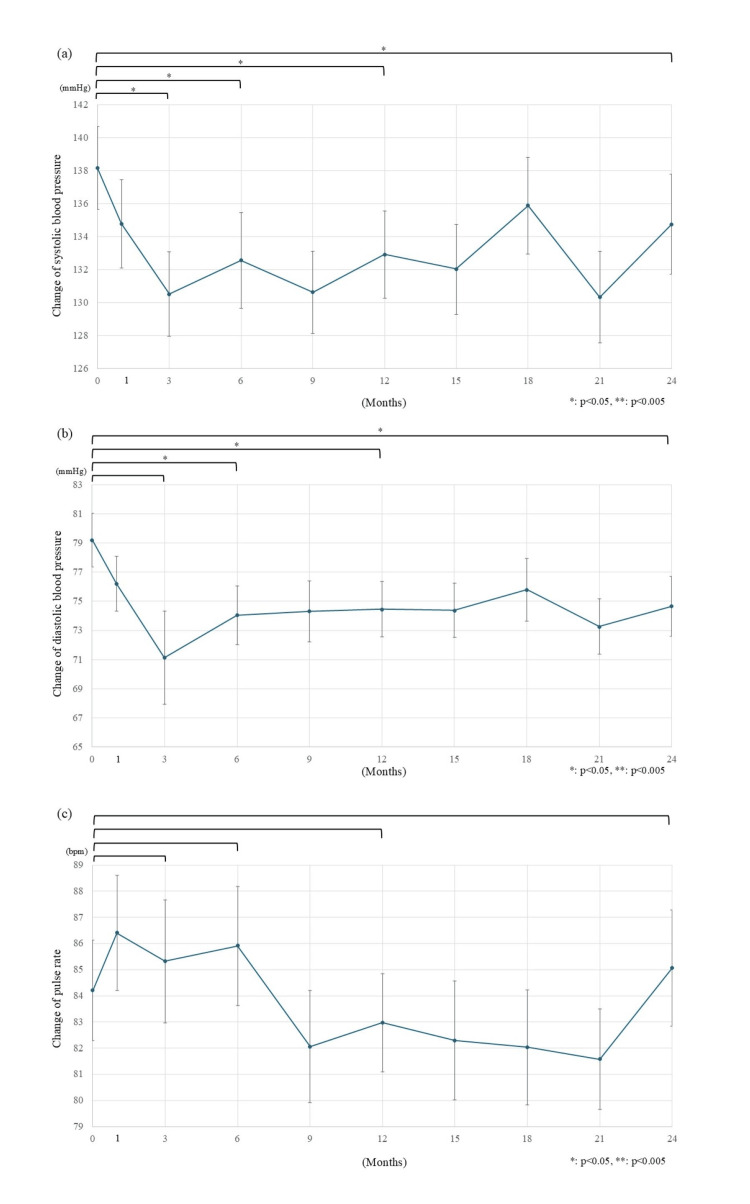
Changes in blood pressure and pulse rate. a) Systolic blood pressure gradually decreased after the start of luseogliflozin. The changes from the baseline were statistically significant, with -5.3 mmHg (from 138.2 mmHg to 132.9 mmHg) after 1 year and -3.4 mmHg (to 134.8 mmHg) after 2 years. b) Diastolic blood pressure decreased after the start of luseogliflozin. The changes from the baseline were statistically significant, with -4.7 mmHg (from 79.2 mmHg to 74.5 mmHg), 1 year after the start of luseogliflozin and -4.5 mmHg (to 74.7 mmHg) after 2 years. c) No statistically significant changes in pulse rate were observed two years after the start of luseogliflozin. The changes from the baseline were -1.2 bpm (from 84.2 bpm to 83.0 bpm) after 1 year and +0.9 bpm (to 85.1 bpm) after 2 years. bpm: beats per minute; The error bars show standard error, *: p<0.05; **: p<0.005

The changes in SBP, DBP, and PR are shown in Figure 4. SBP decreased from 138.2 mmHg to 130.5 mmHg (-7.7 mmHg) after three months from the initiation of luseogliflozin, continuing to change to 132.9 mmHg (-5.3 mmHg) after 1 year and to 134.8 mmHg (-3.4 mmHg) after 2 years (p=0.012, 0.035, and 0.023 respectively) (Figure 4a). DBP decreased from 79.2 mmHg to 71.1 mmHg (-8.1 mmHg, p=0.058) after three months, to 74.5 mmHg (-4.7 mmHg, p=0.016) after 1 year, and to 74.7 mmHg, (-4.5 mmHg, p=0.019) after 2 years (Figure 4b). In contrast, there was no statistically significant change in pulse rate after the start of luseogliflozin treatment (Figure 4c).

## Discussion

SGLT2is were first launched in Japan in 2014, and its market share has increased rapidly since then. However, evidence for their effects is insufficient, and the long-term effects of luseogliflozin, an SGLT2i, have not been established. In this study, we examined the effects of luseogliflozin on glucose metabolism and related anthropometrics over two years in individuals with T2D.

Glycemic control

The statistically significant decreases in RPG and HbA1c indicated the durability of luseogliflozin’s glucose-decreasing effect over two years. Its effects on glycemic improvement were comparable with that of empagliflozin (-0.77% to -0.83%) and canagliflozin (-0.99% to -1.04%) when percent-reduction of HbA1c was employed as a denominator [16,17].

Body weight reduction

During the two years of luseogliflozin administration, BW and BMI substantially decreased by 2.48 kg and 0.88 kg/m^2^ after 1 year and 2.90 kg and 1.03 kg/m^2^ after 2 years, respectively. On BW changes in patients with type 2 diabetes, Looker et al. reported a natural change in BMI of -0.61±0.22 kg/m^2 ^per year [18]. On the other hand, Chaudhry et al. reported that patients using insulin and SU gained 0.44 to 0.42 kg of BW and were treated with BG and 0.24 kg of weight loss per year [19]. In this study, weight loss was observed immediately after treatment initiation, with a reduction of over 2 kg in body weight and nearly 1 kg/m² in BMI during the first year. These changes were greater than those typically seen in the natural course of treatment, as reported in previous studies. Therefore, this change in body weight was considered to be an effect of luseogliflozin. The decrease in BW and BMI was comparable to the values reported for Japanese patients with diabetes after treatment with empagliflozin (-2.78 kg after 1 year and -3.28 kg after 2 years) [16]. The effect of canagliflozin on BW in one year was reported to decrease from 2.94% to 4.42% [17]. The mean BW of the patients in this study was 73.17 kg. Applying this value to the percentage of weight change reported earlier, the amount of weight change was estimated to be from 2.15-3.23 kg, which was equivalent to that previously reported [16,17].

Blood pressure effect

Statistically significant changes in SBP and DBP were observed (-5.3 mmHg, p=0.035 and -4.7 mmHg, p=0.019, respectively) 1 year after the start of luseogliflozin. These effects on BP were greater than that of empagliflozin (-3.8 mmHg reduction in SBP and -2.0 mmHg reduction in DBP) [16]. The decrease in BP caused by canagliflozin was -2.84 to -7.78 mmHg for SBP and -1.90 to -4.44 mmHg for DBP. Although statistical comparisons were not feasible, it was suggested that the effect of luseogliflozin on BP was greater than that of empagliflozin and equivalent to that of canagliflozin [16,17]. BP becomes a direct load on the cardiovascular system and, as a result, is involved in the development of atherosclerosis [20,21]. It is also known that atherosclerosis progresses in patients with T2D [22], and the importance of controlling BP has been emphasized [23]. However, patients with T2D often have multiple comorbidities, such as dyslipidemia as well as hypertension, particularly in older patients [24]. As a result, the issue of polypharmacy has attracted attention in recent years [25]. From this perspective, SGLT2is can be highly beneficial if they contribute not only to blood glucose management but also to BP control. Among them, the strength of luseogliflozin lies in its potential to provide a stronger antihypertensive effect than other SGLT2is.

Limitations

This study had limitations. First, this was a retrospective single-arm observational study. Although the findings of this study can be analyzed in detail, its causes are difficult to determine. Second, this was a single-center study; therefore, whether this phenomenon is unique to this facility or is universal remains unclear. Third, our sample size was small; therefore, examining other factors potentially involved in this phenomenon was difficult. Fourth, in most patients, luseogliflozin was also used as the second or more advanced hypoglycemic agent; because of this, the observed changes were not entirely due to the effect of luseogliflozin. Fifth, in this study, the measurements of BP were single without standardized timing or consideration of pre-measurement activities. As a result, it cannot be ruled out that there is a bias in the fluctuations in blood pressure in this study. Further studies are needed to confirm these findings.

In summary, we thought that luseogliflozin was as effective as other SGLT2is. This suggests that luseogliflozin may have beneficial effects on cardiovascular and renal outcomes, as shown in the SGLT2i meta-analysis [26]. We believe that further study is necessary on this point.

## Conclusions

Luseogliflozin, an SGLT2 inhibitor, maintained improved glycemic control and weight loss for at least two years in clinical practice. This effect was almost the same as that reported for other SGLT2 inhibitors (empagliflozin and canagliflozin). These results suggest that luseogliflozin has equivalent effects on glucose control, BW, and circulation system (BP and PR) as other SGLT2 inhibitors.
